# Extremism, religion and psychiatric morbidity in a population-based sample of young men

**DOI:** 10.1192/bjp.bp.116.186510

**Published:** 2016-12

**Authors:** Jeremy W. Coid, Kamaldeep Bhui, Deirdre MacManus, Constantinos Kallis, Paul Bebbington, Simone Ullrich

**Affiliations:** **Jeremy W. Coid**, MBChB, MD(Lond), FRCPsych, MPhilDipCriminol, Violence Prevention Research Unit, Wolfson Institute of Preventive Medicine, Queen Mary University of London, London; **Kamaldeep Bhui**, MBBS, MSc, MD, FRCPsych, Centre for Psychiatry, Wolfson Institute of Preventive Medicine, Queen Mary University of London, London; **Deirdre MacManus**, MB ChB, MSc, MRCPsych, PhD, Institute of Psychiatry, Psychology and Neurosciences, King's College London, London; **Constantinos Kallis**, PhD, Violence Prevention Research Unit, Wolfson Institute of Preventive Medicine, Queen Mary University of London, London; **Paul Bebbington**, PhD, FRCP, FRCPsych, Division of Psychiatry, Faculty of Brain Sciences, University College London, London; **Simone Ullrich**, PhD, Violence Prevention Research Unit, Wolfson Institute of Preventive Medicine, Queen Mary University of London, London, UK

## Abstract

**Background**

There is growing risk from terrorism following radicalisation of young men. It is unclear whether psychopathology is associated.

**Aims**

To investigate the population distribution of extremist views among UK men.

**Method**

Cross-sectional study of 3679 men, 18–34 years, in Great Britain. Multivariate analyses of attitudes, psychiatric morbidity, ethnicity and religion.

**Results**

Pro-British men were more likely to be White, UK born, not religious; anti-British were Muslim, religious, of Pakistani origin, from deprived areas. Pro- and anti-British views were linearly associated with violence (adjusted odds ratio (OR) = 1.51, 95% CI 1.38–1.64, *P*<0.001, adjusted OR = 1.33, 95% CI 1.13–1.58, *P*<0.001, respectively) and negatively with depression (adjusted OR = 0.72, 95% CI 0.61–0.85, *P*<0.001, adjusted OR = 0.64, 95% CI 0.48–0.86, *P* = 0.003, respectively).

**Conclusions**

Men at risk of depression may experience protection from strong cultural or religious identity. Antisocial behaviour increases with extremism. Religion is protective but may determine targets of violence following radicalisation.

Threats from international terrorism are increasing^1^ with concern over radicalisation and recruitment of young Muslim men. Migrants from countries without effective governance may not easily assimilate and may become radicalised, with extremist views. However, extremist views may also involve nationalism. In both contexts, war may be perceived as a ‘just cause’. There has been little research into factors maintaining loyalty and willingness to fight for a country, and pathways to terrorism remain complex and unclear. However, potential recruits for terrorist groups are more likely to feel angry, alienated or disenfranchised, believe political involvement cannot effect change, identify with perceived victims of social injustice and feel need to take action. They believe violence against the state is justified, have friends or family sympathetic to the cause, and benefit psychologically from sense of adventure, camaraderie and heightened identity.^2^ Poor mental health could be associated with some of these characteristics, increasing risk of engaging in terrorism or becoming radicalised. A common characteristic, however, is that terrorists, unless lone actors,^3^ have little evidence of psychopathology.^4–7^ Severe mental disorders are therefore not commonly associated with terrorism, but mental disorder may confer vulnerability to radicalising influences.^8^ Extremism could be a reaction to adversity, leading to empowerment, overcoming or averting symptoms such as depression. By 2010, UK media reported growing numbers of British-born Muslim men, mainly of Pakistani descent, recruited by Al-Qaeda, trained in Pakistan and fighting against the British army in Afghanistan. This represented open armed conflict against British political and cultural values. It is unclear whether these men were an isolated minority or came from communities with shared attitudes supporting terrorism. Support for terrorism within a population is thought to resemble a pyramidal structure running from the largest proportion at the bottom, consisting of neutral individuals, and moving up through levels of diminishing size, from sympathisers, to supporters, with finally terrorists at the apex.^4,9^

No previous research has investigated the population distribution of extremist attitudes and support for armed conflict and corresponding associations with mental disorder. We therefore carried out a representative survey of the attitudes of men aged 18–34 years in England, Scotland and Wales towards the war in Afghanistan. Our aims were to investigate: (a) the population distribution of pro-British and anti-British views associated with the war; (b) linear associations between ethnicity/religion, violence/criminality and psychiatric morbidity; and (c) explanatory factors for observed associations.

## Method

### Data collection

The survey was carried out in 2011 and has previously been described.^10^ It was based on random location sampling, an advanced form of quota sampling shown to reduce biases introduced when interviewers are able to choose locations to sample from. Individual sampling units (census areas of 150 households each) were randomly selected within British regions in proportion to their population. The basic survey included a representative sample of young men (18–34 years) from England, Scotland and Wales. In addition, there were two boost surveys. First, young Black and minority ethnic (BME) men were selected from output areas with a minimum of 5% BME inhabitants. Second, young men from lower social grades (as defined by the market research society and based on head of household: semi-skilled, unskilled, occasional manual workers; and pensioners and welfare recipients) were selected from output areas in which there was a minimum of 30 men 18–34 years of age in these social grades. A self-administered questionnaire piloted in a previous survey was adapted for this study. Informed consent was obtained from all survey respondents, who then completed pencil and paper questionnaires in privacy. They were paid £5 for participation. Ethical approval to carry out the survey was granted by the Ethics Committee, Queen Mary University of London, UK.

### Survey measures

The Psychosis Screening Questionnaire^11^ screened participants for psychotic experiences; individuals were defined as screen-positive if they met three or more criteria. Questions from the Structured Clinical Interview for DSM-IV personality disorders screening questionnaire^12^ identified antisocial personality disorder. Anxiety disorder and depression were defined as a score of ⩾11 on the Hospital Anxiety and Depression Scale^13^ in the past week. Scores ⩾20 on the Alcohol Use Disorders Identification Test^14^ and ⩾25 on the Drug Use Disorders Identification Test^15^ indicated alcohol dependence and drug misuse disorder, respectively. Participants were questioned about violent behaviour as in previous surveys of violence.^16^ Information was sought about involvement in and attitudes towards violence.

We could not ask participants if they actively supported terrorism. We used proxy measures to investigate extremist attitudes, support for Britain and the war in Afghanistan. We asked which of the following applied: ‘I feel strongly British (English, Scottish, Welsh or Northern Irish) if that means standing up for yourself or your country’; ‘I feel more like people with my own religious, cultural or political beliefs than people who are British’; ‘I support the war in Afghanistan’; ‘I oppose the war in Afghanistan’; ‘I could fight in the British army in Afghanistan’; ‘I could fight against the British army in Afghanistan’. The variable pairs were combined to reflect extremes in both directions. There were few differences between those who responded negatively to each pair of questions and those who endorsed ‘I don't know’. These two groups were therefore combined and constituted the reference group against which the extremes were contrasted.

To study the distribution of degrees of extremism we created two exposure variables: ‘pro-British’ and ‘anti-British’ by combining the variables described above. Those who endorsed negative answers or ‘don't know’ to cultural identity, support of/opposition to the war, and fighting for/against the British army were coded ‘0’ and constituted the reference group for both variables. Study participants who reported British/dual identity or supported the war or own identity or opposed the war received a rating of ‘1’. Those who reported British/dual identity and supported the war or own identity and opposed the war received a rating of ‘2’. A rating of ‘3’ was assigned when the person reported support for, or opposition to, the war in Afghanistan. The higher the rating on these two variables, the stronger were the opinions towards the extreme. The survey participants were also asked about current religious affiliation, how often they attended religious services in the past month, and how often they prayed.

### Statistical analysis

For descriptive purposes, weighted absolute and relative frequencies were reported for binary/polytomous variables and weighted means and standard deviations for variables on interval/ratio level. We ran weighted survey commands for logistic regression models with binary outcomes. Analyses incorporated adjustment of standard errors for clustering of individuals within postcodes and important demographic characteristics (age and index of multiple deprivation).

We first studied associations between the three exposure variables cultural identity, support for/opposition to the war, and fighting for/against the British army. We then investigated whether increasing extremist views in both directions were related to demography, ethnicity, religion, psychiatric morbidity and violence and criminal behaviour. The two variables (pro-British and anti-British) were treated as continuous exposure in the logistic regression model.

In a final step we investigated psychiatric vulnerabilities and protective effects in ethnic minority and religious groups and associations with religiosity. If (a) a significant association between ethnic/religious group and psychiatric morbidity was found and (b) if the ethnic/religious group also demonstrated a significant relationship with extremism, and (c) if extremism was associated with psychiatric morbidity, we included the extremism variables in the statistical model. If the association between ethnic/religious group and psychiatric illness was no longer significant following adjustment, extremism was interpreted as an explanatory variable in this relationship.

All statistical analyses were carried out using Stata 14. A significance level of <0.05 was adopted throughout.

## Results

### Demography and sampling

The weighted sample included 3679 men aged 18–34 years: 1983 (53.9%) from the main survey; 1075 (29.2%) from the minority ethnic sample; and 621 (16.9%) from the sample of men from lower social classes. Their mean age was 25.9 years (s.d. = 5.0), more than half were single (2232, 61.5%) and more than a third were unemployed (1382, 38.6%). The sample was ethnically diverse, with 38.5% (1413) participants of BME origin.

### Associations between cultural/national identity, support for/opposition to the war, and fighting for/against the British army

When contrasted to those endorsing ‘no’ or ‘I don't know’, men reporting British identity demonstrated higher rates of support (adjusted OR = 5.38, 95% CI 3.88–7.47, *P*<0.001) and opposition to the war (adjusted OR = 2.37, 95% CI 1.80–3.10, *P*<0.001). This was also true of men identifying with their own culture, ethnicity or cultural group (adjusted OR = 1.91, 95% CI 1.05–3.49, *P* = 0.034 for support; adjusted OR = 4.40, 95% CI 2.98–6.49, *P*<0.001 for opposition). The pattern was similar among those identifying with both cultures (adjusted OR = 3.41, 95% CI 2.27–5.12, *P*<0.001 and adjusted OR = 2.52, 95% CI 1.71–3.71, *P*<0.001, respectively).

Men with strong British identity and those identifying with both cultures were more likely to report that they would fight in the British army (adjusted OR = 4.23, 95% CI 3.20–5.59, *P*<0.001 and adjusted OR = 4.07, 95% CI 2.60–6.38, *P*<0.001, respectively) but not against it. In contrast, men identifying uniquely with their own culture were more likely to report they would fight against the British army (adjusted OR = 5.08, 95% CI 1.97–13.10, *P*<0.001) but not in it. Among men with British identity, support for the war was significantly associated with willingness to fight in the British army (adjusted OR = 5.48; 95% CI 3.11–9.64, *P*<0.001). Among men who identified with their own culture, opposition to the war was associated with willingness to fight against the British army (adjusted OR = 4.74, 95% CI 1.07–88.38, *P* = 0.043).

### The epidemiology of extremism

**Fig. 1:**
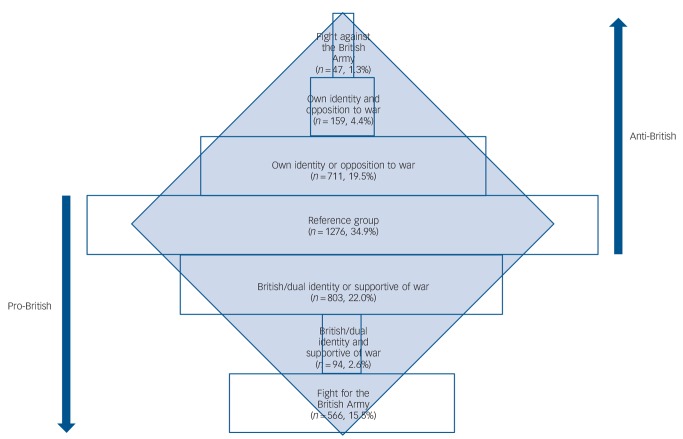
The distribution of extremism among young British men.

The distribution of increasing degrees of extremism in opposing directions (pro-British and anti-British) is shown in Fig. 1. Approximately a third were neutral, answering all questions either negatively or endorsed ‘don't know’ (reference group for both extremes). The distribution towards the anti-British extreme followed closely the shape of a pyramid. The shape differed towards the pro-British extreme where more substantial numbers of study participants endorsed willingness to fight in the British army (pro-British extreme).

Associations between these opposing trends and demographic characteristics are shown in online Table DS1 and those for psychiatric morbidity, violence and crime are shown in Table 1. Trends towards the pro-British extreme demonstrated significant inverse relationship with higher educational qualifications, most ethnic minority groups including Muslim, Hindu, Jewish/Sikh/Buddhist/other, attendance at services, frequency of praying (online Table DS1) and depressive illness (Table 1). There were significant positive associations with drug misuse, antisocial personality disorder and all variables measuring violence and crime.

**Table 1:** Attitudes towards both extremes, psychiatric morbidity and violence^a^

	Pro-British		Anti-British	
	Adjusted OR (95% CI)	*P*	Adjusted OR (95% CI)	*P*
Psychopathology				
Psychosis	1.12 (0.90–1.39)	0.302	0.92 (0.51–1.67)	0.795
Anxiety	0.92 (0.81–1.05)	0.222	0.78 (0.60–1.02)	0.067
Depression	0.72 (0.61–0.85)	<0.001	0.64 (0.48–0.86)	0.003
Alcohol dependence	1.08 (0.93–1.25)	0.324	0.77 (0.52–1.16)	0.217
Drug misuse	1.16 (1.04–1.29)	0.008	1.13 (0.86–1.48)	0.388
Antisocial personality disorder	1.52 (1.36–1.69)	<0.001	1.54 (1.22–1.94)	<0.001

Violence and crime				
Any violence in past 5 years	1.51 (1.38–1.64)	<0.001	1.33 (1.13–1.58)	<0.001
Excited by violence	1.40 (1.20–1.63)	<0.001	1.40 (0.93–2.11)	0.110
Criminal conviction (any)	1.46 (1.31–1.63)	<0.001	1.02 (0.80–1.30)	0.885
In prison (ever)	1.55 (1.27–1.89)	<0.001	1.81 (1.29–2.52)	<0.001

a. See online Table DS1 for data relating to demographic characteristics, ethnicity and religion. Logistic regression adjusted for survey type, age and index of multiple deprivation. 95% confidence intervals were estimated using robust standard errors to account for correlations within survey areas because of clustering within postcodes.

Trends towards anti-British extremism were positively associated with socioeconomic deprivation, other White, Indian, Pakistani, Bangladeshi, Black African, Chinese/other ethnicity, non-UK born, other Christian, Muslim, Jewish/Sikh/Buddhist/other religion, attendance at services and frequency of praying (online Table DS1). Increasing anti-British attitudes demonstrated positive relationships with antisocial personality disorder, violence and imprisonment and an inverse association with depression (Table 1).

### Ethnicity, religion, and psychiatric morbidity

We then investigated associations between ethnicity, religion and psychiatric morbidity (Table DS2). Ethnicity and religion/religiosity were not associated with psychosis or anxiety disorder, apart from an inverse association between Protestant religion and anxiety disorder. The prevalence of depression was significantly higher among Pakistani and Black minority groups than UK-born White men. Alcohol dependence was less prevalent among Pakistani men, all religious groups and among those endorsing measures of religiosity compared with those affirming no religion/atheism. Contrasted with White British men, there was less drug misuse among Indian, Pakistani, Bangladeshi and Chinese/other minority groups. Compared with men who were not religious, there was less drug misuse among all religious groups and degrees of religiosity, except Jewish/Sikh/Buddhist/other who showed no difference. Antisocial personality disorder was also less prevalent among Indian, Pakistani and Bangladeshi men, Protestant, Muslim and Hindu men and among those endorsing religiosity.

### Explanatory analyses

In a final step, we investigated whether associations found between ethnic/religious groups and psychiatric morbidity were explained by extremist views, both pro- and anti-British. Ethnic and religious groups listed in Table 2 were selected on the basis of associations with extremist views (Table 1 and online Table DS1) and psychiatric morbidity (online Table DS2), which in turn were associated with extremist views (Table 1 and online Table DS1). Most associations between ethnicity and religious groups remained significant after adjustment for extremism variables and appeared to exert direct effects. Explanatory effects of extremist views were found for depression among Pakistani men; drug misuse among Indian and other Christian men; antisocial personality disorder among Indian and Hindu young men. This indicated that negative associations between these minority/religious groups and psychiatric morbidities were not direct but were explained by extremist views.

**Table 2:** Explanatory model: ethnic/religious groups and their association with psychiatric morbidity^a^

	Depression – baseline		Depression – adjusted		Drug misuse – baseline		Drug misuse – adjusted		Antisocial personality disorder – baseline		Antisocial personality disorder – adjusted	
	Adjusted OR^b^ (95% CI)	*P*	Adjusted OR^c^ (95% CI)	*P*	Adjusted OR^b^ (95% CI)	*P*	Adjusted OR^c^ (95% CI)	*P*	Adjusted OR^b^ (95% CI)	*P*	Adjusted OR^c^ (95% CI)	*P*
Minority ethnic group												
Indian	n/a		n/a		0.49 (0.24–0.97)	0.042	0.62 (0.27–1.40)	0.250	0.25 (0.10–0.61)	0.002	0.41 (0.15–1.13)	0.085
Pakistani	2.26 (1.23–4.16)	0.009	1.88 (0.85–4.15)	0.118	0.28 (0.13–0.61)	0.001	0.23 (0.08–0.62)	0.004	0.19 (0.09–0.42)	<0.001	0.19 (0.08–0.46)	<0.001
Bangladeshi	n/a		n/a		0.25 (0.09–0.66)	0.005	0.10 (0.02–0.56)	0.009	0.04 (0.01–0.31)	0.002	0.06 (0.01–0.47)	0.007
Black African	2.59 (1.29–5.17)	0.007	2.71 (1.16–6.30)	0.021	n/a		n/a		n/a		n/a	
Chinese/other	n/a		n/a		0.39 (0.15–0.98)	0.044	0.35 (0.12–1.04)	0.060	0.33 (0.13–0.85)	0.021	0.30 (0.10–0.94)	0.039

Religion/religiosity												
Other Christian	n/a		n/a		0.41 (0.23–0.73)	0.002	0.49 (0.24–1.01)	0.052	n/a		n/a	
Muslim	n/a		n/a		0.21 (0.11–0.38)	<0.001	0.22 (0.11–0.46)	<0.001	0.26 (0.15–0.46)	<0.001	0.30 (0.15–0.60)	0.001
Hindu	n/a		n/a		0.21 (0.08–0.54)	0.001	0.07 (0.01–0.53)	0.011	0.36 (0.14–0.90)	0.029	0.43 (0.13–1.42)	0.165

Attendance at services	n/a		n/a		0.71 (0.61–0.84)	<0.001	0.78 (0.68–0.89)	<0.001	0.78 (0.69–0.88)	<0.001	0.84 (0.75–0.95)	0.006

Frequency of praying	n/a		n/a		0.72 (0.61–0.85)	<0.001	0.75 (0.66–0.86)	<0.001	0.82 (0.75–0.90)	<0.001	0.84 (0.75–0.95)	0.004

n/a, not applicable.

a. Ethnic and religious groups were selected Pased on significant associations with both extremism and psychiatric morbidity. Psychiatric morbidities were selected because of their significant relationship with extremism.

b. Logistic regression adjusted for survey type, age and index of multiple deprivation.

c. Further adjustment for extremism variables. 95% confidence intervals were estimated using robust standard errors to account for correlations within survey areas because of clustering within postcodes.

## Discussion

### Population distribution of extremism

We are not aware of a previous population study that has investigated the distribution of extremist attitudes and associations with psychiatric morbidity. We showed that identification with British national or other cultural identity did not determine direction of support or opposition to the war but were associated with holding strong opinions. Men could have a strong British identity yet oppose the war, and vice versa. However, a combination of strong British identity and support for the war were associated with willingness to fight in the army; other cultural identity and opposition to the war with willingness to fight against Britain. We next investigated the distribution of these attitudes and confirmed a pyramidal structure within this population, corresponding to a postulated pyramidal model of distribution of support for terrorism.^4,9^ A large number of men held neutral views at the base of this structure, progressing through increasing levels of opposition to the war and non-British identity, with willingness to fight against the British army at the apex (Fig.1). However, when we attempted to apply this structure to the larger group of men who supported their country, the pyramidal distribution (inverted) was not closely replicated. Despite declining support for the war by 2011, 1 in 6 young adult British men still reported they would fight in the army.

The stronger their pro-British attitudes, the more likely men were White and UK-born, but less likely to report higher educational qualifications or religious affiliation. They were more likely to report violence in the past 5 years, excitement from violence, criminal convictions and imprisonment. With increasing anti-British attitudes, men were more likely to be non-UK born, living in socioeconomically deprived geographical areas, from BME subgroups and religious. Nevertheless, they were similar to those with pro-British attitudes in being more likely to report violence and imprisonment.

Externalising and antisocial behaviour are not uncommon among applicants for military service in the USA and European countries.^17,18^ Our findings correspond with recent trends in recruitment to terrorist organisations, with decline in education and socioeconomic status,^6^ and need for those prepared to engage in open armed conflict rather than covert operations requiring specialist skills and subterfuge. They also correspond to successful targeting of prison populations for radicalisation and recruitment.^19^

### Extremism and depression

The key finding was that men at the bottom of this structure, with neutral or undecided views, (Fig. 1) were more likely to be depressed. Anti-British extremist views may have offered protection against depression, specifically among men of Pakistani origin. These findings correspond to the hypothesis that lack of personal identity and meaning, with unfulfilled need for belonging, create psychological vulnerability both to extremism^8,20–22^ and anxiety and depression.^23^ Within this theoretical framework, attributing blame, identifying responsible perpetrators,^24^ strong national or other cultural identity, and active support for or opposition to a cause, may protect against depression. For some men, depression may be a precursor to ‘mobilisation’, leading to active support for and consideration of involvement in terrorism or armed conflict along a pathway of radicalisation.^8^ Lack of identity and uncertainty, together with depression, may contribute to a vulnerable state in which personal crisis can act as a trigger, resulting in an opening for new beliefs and values,^25–27^ encouraged by people holding similar values that legitimise violence.^28^ Relatives' and friends' experiences of social exclusion, including poverty and reported experiences of racism, may have influenced these individuals to take a more active position. Factors such as turning to religion or new political beliefs triggered by a war (against people with similar cultural and religious characteristics) could result in a protective sense of empowerment involving new meaning, belief systems and identity^25^ along a pathway ultimately leading to violent action. However, since we cannot determine the direction of association in this cross-sectional survey, respondents with depression may simply have been less likely to fight for or against their country or to hold extreme views because of their depression.

The other key finding observed towards opposite extremes in Fig. 1 was a linear association with antisocial personality disorder and violence. Decisions to fight for or against a country may involve a process of selection. Those with pre-existing antisocial and pro-violent dispositions may simply be more likely to hold extreme attitudes and be more suited for military action than those whose attitudes are neutral or who are depressed.

### Religion

As expected, religious affiliation and practice was protective against externalising disorders such as alcohol dependence, drug misuse and antisocial personality disorder, in line with religious teachings.^29,30^ Increasing anti-British extremist views were linearly associated with religion and religiosity, whereas increasing support for British intervention in Afghanistan was negatively associated. This polarisation of religious views may reflect ongoing processes of mutual rejection and an exclusionary circle in the British population, currently evident among many Muslim and non-Muslim communities.^31^ Within these communities, particularly those with multiple risk factors for radicalisation, extremism may result from perception of an aggressive, oppressive non-Muslim culture. A four-stage model of Al-Qaeda-influenced radicalisation proposes a pre-radicalisation stage of vulnerability followed by early exploration of Salafi Islam and gradual gravitation away from old identity, association with like-minded individuals and adoption of ideology.^24^ This is followed by progressive adoption of jihadi-salafi ideology and finally jihadisation.^6^ Further investigation is needed into whether depression is intensified among Muslims by this mutual exclusionary process and whether radicalisation, with new sense of belonging and identity,^32^ and willingness to take violent action are associated with protection and symptomatic improvement.

Experimental studies of support for suicide attacks have found that attendance at religious services corresponds as a function of collective religious activities, but differed from our findings in that frequency of praying does not.^33^ We did not investigate support for suicidal attacks. Furthermore, these studies found inconsistencies across different countries and that these specific associations with religious practice were strongest among Palestinians and Israeli settlers. In the UK, needs for excitement through violent action, camaraderie and strong masculine identity may, for some men, be more important proximal factors than religion, politics or cultural identity. Nevertheless, the latter may legitimise and ultimately determine targets of violence.

### Limitations

Our survey had several limitations, including our method of defining identity where we combined ethnicity, cultural, religious and political beliefs. Although we defined support for armed conflict as ‘extremism’ for the purpose of our study, it could not be ascertained whether men reporting willingness to fight against the British Army actually intended to do so. Violent behaviour within the past 5 years was self-reported, without objective information such as data on arrest or convictions. Use of self-report captures more violence than comes to the attention of the authorities but may still have underestimated true prevalence. Socially undesirable behaviours may be underreported because of religious beliefs and cultural expectations.

Clinical presentations were derived from self-report questionnaires and not confirmed by clinical interview. However, clinician assessments compare favourably with self-report instruments. Given the cross-sectional nature of the data, we could not identify whether these measures were associated with changes in cultural, ethnic, religious and political views held by the respondent. Nonetheless, the community-based design and large sample size allowed examination of associations between different population subgroups holding different beliefs and mental disorder categories. Furthermore, sample size provided sufficient statistical power to test complex models and control for confounding.

Random location sampling does not provide detailed information on number of young men who declined to participate. However, because the method is based on the national census, participants were identified and included according to representative strata and their actual frequency in the population. Because young adult men of lower social class are more likely to decline participation in household surveys, this method has considerable advantages when investigating antisocial behaviour. The alternative would be to rely on a method requiring statistical weighting to adjust for attrition, which may be particularly high among this subgroup.

The finding that anti-British extremist views were negatively associated with depression among Pakistani men but not Black African men may be as a result of heterogeneity among countries included in the latter combined category. The BME boost sample, although large and representative, was underpowered to examine men from specific African countries with large Muslim populations and ongoing insurgencies. It was of interest that men of Bangladeshi origin were no more likely to hold anti-British extremist views or to have depression than White UK-born men. However, it would be necessary to conduct further studies with larger BME samples to investigate the roles of differing experiences of racial discrimination, social mobility and ethnic density effects across certain BME subgroups. It would also be necessary to investigate the effects of right-wing and anti-immigrant views on exacerbation of mutual rejection and an exclusionary circle.^31^

### Implications

Since withdrawal of coalition forces from Afghanistan, concern has grown over future support for terrorism within BME communities, particularly in Europe. Young men who travel to fight for Islamic State in Syria and Iraq, follow radical interpretations of Islam, and oppose political and economic interests of their host countries may return to threaten national security through insurgency. Religious identity may have become an increasingly important unifying and mobilising factor since 2011, when shared language, geographical and cultural origin, and blood ties additionally contributed to willingness to fight against the British army in southern Afghanistan among young men of Pakistani origin.^34^ Our findings indicated considerable heterogeneity across the population among those expressing extremist views, with deep divisions in personal attitudes and identity corresponding to processes of mutual rejection and exclusion. Depressive symptoms are more prevalent among minority populations in Europe.^35^ Separation with maintenance of ethnic culture but no participation in local culture is associated with mood and anxiety disorders.^36^ Further research is needed into interventions that reduce discrimination, exclusion and extremism in these communities and associated depression.

Extremism can spread to those with moderate views through epidemic transmission when culture, religious and political values shift quickly.^37^ Views and opinions initially considered extreme may become the norm.^38^ Simulated models suggest that when a large part of the population hold moderate views or are uncertain, extreme views tend to prevail. This leads to either convergence on a single extreme or to bipolarisation.^39^ Bipolarisation implies strongly opposing views with neither achieving dominance.

Endorsement of willingness to fight for one's country would not be interpreted by most UK people as ‘extremism’ but willingness, if necessary, to make an extreme sacrifice for the benefit of, and to defend, the majority. Fusion theory would suggest that the asymmetrical distribution in Fig. 1 was explained by more UK men being strongly ‘fused’ through their personal and social identity with UK values to make such sacrifices.^40^ However, this process is thought to depend on whether members of a group share the same core characteristics and values. Individuals in the group are more likely to fight and die for family members, or those with whom they perceive close ties similar to those in families within small social groups than for their country. Promoting shared values and perceived similarity in personal identity may therefore be important in preventing processes that lead to bipolarisation. Our study may be a starting point for more sophisticated investigation into whether these processes are failing in European countries, whether bipolarisation has accelerated and intensified within vulnerable communities with high levels of depression, leading to convergence on a single extreme, and increasing numbers of radicalised young men prepared to engage in armed conflict.
